# The Effect of Noisy Galvanic Vestibular Stimulation on Learning of Functional Mobility and Manual Control Nulling Sensorimotor Tasks

**DOI:** 10.3389/fnhum.2021.756674

**Published:** 2021-11-03

**Authors:** Esther J. Putman, Raquel C. Galvan-Garza, Torin K. Clark

**Affiliations:** ^1^Ann and H.J. Smead Aerospace Engineering Sciences, University of Colorado, Boulder, Boulder, CO, United States; ^2^Lockheed Martin Advanced Technology Laboratories, Arlington, VA, United States

**Keywords:** stochastic resonance, white noise, retention, locomotion, orientation perception

## Abstract

Galvanic vestibular stimulation (GVS) is a non-invasive method of electrically stimulating the vestibular system. We investigated whether the application of GVS can alter the learning of new functional mobility and manual control tasks and whether learning can be retained following GVS application. In a between-subjects experiment design, 36 healthy subjects performed repeated trials, capturing the learning of either (a) a functional mobility task, navigating an obstacle course on a compliant surface with degraded visual cues or (b) a manual control task, using a joystick to null self-roll tilt against a pseudo-random disturbance while seated in the dark. In the “learning” phase of trials, bilateral, bipolar GVS was applied continuously. The GVS waveform also differed between subjects in each task group: (1) white noisy galvanic vestibular stimulation (nGVS) at 0.3 mA (2) high-level random GVS at 0.7 mA (selected from pilot testing as destabilizing, but not painful), or (3) with the absence of stimulation (i.e., sham). Following the “learning” trials, all subjects were blindly transitioned to sham GVS, upon which they immediately completed another series of trials to assess any aftereffects. In the functional mobility task, we found nGVS significantly improved task learning (*p* = 0.03, mean learning metric 171% more than the sham group). Further, improvements in learning the functional mobility task with nGVS were retained, even once the GVS application was stopped. The benefits in learning with nGVS were not observed in the manual control task. High level GVS tended to inhibit learning in both tasks, but not significantly so. Even once the high-level stimulation was stopped, the impaired performance remained. Improvements in learning with nGVS may be due to increased information throughput resulting from stochastic resonance. The benefit of nGVS for functional mobility, but not manual control nulling, may be due to the multisensory (e.g., visual and proprioceptive), strategic, motor coordination, or spatial awareness aspects of the former task. Learning improvements with nGVS have the potential to benefit individuals who perform functional mobility tasks, such as astronauts, firefighters, high performance athletes, and soldiers.

## Introduction

Galvanic vestibular stimulation (GVS) is a non-invasive tool by which electrical stimulation can be applied to the vestibular system through transcutaneous current applied through electrodes placed at the mastoid processes (behind the ears) (Utz et al., 2010). Previous studies have suggested GVS to be a potential tool for enhancing vestibular performance (Mulavara et al., 2011) and shown improvements in perception of small, passive self-motions (Galvan-Garza et al., 2018). Performance benefits following GVS treatment are often thought to occur due to stochastic resonance (SR), a phenomenon in which the response of a non-linear system to an input signal is benefited by the presence of a particular non-zero level of noise (Aihara et al., 2010). SR is broadly described as “noise benefit” (McDonnell and Abbott, 2009) and this benefit can be represented by a pseudo-bell shape performance curve as a function of the noise level added, with a peak in performance at some optimal noise level (Moss et al., 2004).

While a growing number of studies have demonstrated improvement of sensorimotor performance with GVS, few have explored the potential retention of effects on performance once GVS treatment has been removed. Keywan et al. (2020) found no evidence of improved roll tilt thresholds following GVS treatment. However, this study used passive application of nGVS to seated subjects prior to performing a balance task. A 2016 postural stability study found evidence of balance improvement after cessation of stimulation potentially due to vestibular neuroplasticity (Fujimoto et al., 2016). However, this study included only elderly adults (mean age 66.7 ± 0.4 years) and GVS stimulation was applied either passively between performance evaluations or actively but only during postural sway evaluation. Nooristani et al. (2019) raised concerns about Fujimoto’s study design, namely that the lack of a control group prevents controlling for an ordering confound. In their replication of the Fujimoto study, adding a control group, they found no significant difference in improvement between nGVS and sham groups, suggesting that the improvement seen by Fujimoto et al. (2016) was due to a repeated measure learning effect.

Our study sought to evaluate retention of effects of GVS on performance by applying stimulation during the active learning of a task, rather than passive application before task completion. In doing so, the effect of GVS on both learning and retention of task performance can be explored. We also sought to explore the effect of GVS treatment on learning and retention in more complex and operationally relevant tasks, rather than postural sway. As such, subjects completed either a functional mobility or manual control task.

Functional mobility and manual control are essential performance tasks for both pilots and astronauts, which rely on healthy vestibular function for optimal performance. The future of crewed space exploration will involve long duration deep space missions that pose a cascade of physiological adaptations and potential risks to human health. Deleterious effects of spaceflight may also impair operational performance and task completion. Previous studies show that spaceflight causes an increased risk of vestibular dysfunction and spatial disorientation (Reschke and Clément, 2018; Clark, 2019), which pose significant risks for spacecraft control (Bloomberg, 2015). As such, improving vestibular signal detection and overall performance may assist astronauts to perform such tasks despite spaceflight induced maladaptation. GVS may have benefits for individuals on Earth as well, improving balance and performance, for example, in elderly individuals who otherwise may be at a higher risk for falls (Inukai et al., 2018a). Scientifically, GVS has been shown to impact some aspects of bodily awareness (Ferrè et al., 2013c), somatosensory perception (Ferrè et al., 2013a), and even sense of self (Ferrè et al., 2014), which may be relevant for these types of operational sensorimotor tasks.

In this study, two different applications of GVS during task learning were explored. The impact of noisy galvanic vestibular stimulation (nGVS) treatment on task learning as assessed in both the functional mobility and manual control tasks. nGVS has grown in popularity as a method for improving vestibular performance in areas such as static and dynamic balance (Goel et al., 2015; Stefani et al., 2020), postural sway (Inukai et al., 2018b), locomotor stability (Mulavara et al., 2015), manual control (Galvan-Garza, 2016), and roll tilt vestibular perception in direction recognition tasks (Galvan-Garza et al., 2018; Keywan et al., 2018). However, little research has been done to explore the effect of noisy GVS on the potential enhancement of performance when the treatment is applied during active learning of task or whether enhanced learning with GVS is retained once treatment has been removed.

This study also explored whether a high level disruptive GVS waveform could be used to improve sensorimotor learning. This type of GVS signal can cause postural instability (MacDougall et al., 2006) and has been used to mimic locomotor dysfunction and spatial disorientation (Moore et al., 2006, 2011). High levels of GVS can also impact spatial perception (Ferrè et al., 2013b). Learning in a disruptive vestibular signaling environment may prompt adaptation displayed by rapid learning and increased performance once such disruption has been removed. To our knowledge, no previous studies have explored utilizing high level randomized GVS to impact learning of sensorimotor tasks. Our selected parameters for both nGVS and high GVS conditions can be found in the Methods, and elaboration of those selections as they relate to our results can be found in the Discussion.

Tests of sensorimotor performance and learning typically have substantial inter-individual differences. This was expected to be true of the two sensorimotor tasks in this study as well. However, vestibular perceptual thresholds have been shown to explain individual differences in performance of manual control tasks, including in hypergravity (Rosenberg et al., 2018), as well as balance performance tasks (Bermúdez Rey et al., 2016; Karmali et al., 2017, 2021). Due to its relationship to the manual control task, we assessed roll tilt direction recognition (DR) thresholds in the subjects that subsequently performed manual control testing. These thresholds were then investigated to explore potential correlations between DR threshold and sensorimotor performance and learning on the manual control task.

As a preliminary investigation into the effects of GVS on sensorimotor task *learning*, we explored two GVS waveforms aimed at improving learning of operationally relevant sensorimotor tasks. We hypothesized that nGVS may improve learning of both tasks by improving vestibular information processing, and that this enhanced performance may be retained even when stimulation was removed. We hypothesized that high GVS may be disruptive to performance, but that learning in this disruptive environment may result in improved performance once stimulation was removed. Finally, DR thresholds were explored as a potential method for predicting inter-subject variability on manual control task performance.

## Materials and Methods

This experiment design was approved by the University of Colorado at Boulder Institutional Review Board under protocol #20-0097 and all subjects signed a written informed consent form. Thirty-six healthy subjects were recruited for participation in this study (13F, ages 18–32, mean age 22.7 years). Subjects were prescreened and excluded from the study if they had a history of vestibular dysfunction or if they scored above the 90th percentile on the Motion Sickness Susceptibility Questionnaire (Reason, 1968), as we wanted to avoid the potential for motion sickness during our protocols in highly susceptible individuals. Subjects were randomly assigned to one of three treatment groups: sham GVS, nGVS, or High GVS. Subjects were also randomly assigned to one of two performance tasks: a functional mobility assessment or manual control assessment such that in total, six subject groups were formed (Table 1).

**TABLE 1:** Summary of subject demographics and distribution amongst treatment groups.

**Group**	**Task**	**Treatment**	**Subjects**	**Age (year) mean ± SD**	**Sex**
1	FMT	Sham	6	26.0 ± 5.6	2F
2	FMT	nGVS at 300 μA bipolar 0–30 Hz white noise	6	22.3 ± 2.9	2F
3	FMT	High-level GVS at 700 μA bipolar 1.5 Hz random noise	5	24.6 ± 3.6	1F
4	Manual control	Sham	7	21.3 ± 2.4	2F
5	Manual control	nGVS at 300 μA bipolar 0–30 Hz white noise	7	21.4 ± 2.0	5F
6	Manual control	High-level GVS at 700 μA bipolar 1.5 Hz random noise	5	21.0 ± 2.3	1F

The experimental protocol consisted of two phases: learning trials and aftereffect trials. Learning trials were completed with the GVS treatment as randomly assigned to the subject (this could include sham GVS in which no current was applied). In aftereffect trials, any GVS current was deactivated, such that all subjects experienced the sham GVS treatment. Subjects were blind to the transition in treatment between learning and aftereffects trials.

Galvanic vestibular stimulation was administered using 2 × 2 inch sponge electrodes (Caputron) placed on the mastoid processes. Subjects were prepped by manually exfoliating the skin along the mastoid processes (behind the ears) using NuPrep exfoliant gel. The area was then cleaned using 70% isopropyl alcohol. Sponge electrodes were saturated with 7 ml of 0.9% sodium chloride sterile saline per manufacturer’s instructions. Sponge electrodes were held in place against the mastoid process on each side of the skull and secured using an elastic headband.

The nGVS waveform was identical to that previously used to improve balance (Mulavara et al., 2011; Goel et al., 2015), locomotion (Mulavara et al., 2015), and vestibular perceptual thresholds (Galvan-Garza et al., 2018). Studies have shown high variability in subject optimal levels of noisy GVS stimulation for performance enhancement, with average optimal levels typically ranging between 100 and 500 uA (Iwasaki et al., 2014; Goel et al., 2015; Keywan et al., 2018). It appears that at approximately 300 uA (± peak to peak), most subjects have improved perceptual thresholds (Galvan-Garza et al., 2018), so we chose to use this single nGVS stimulus amplitude rather than attempt to personalize for each subject.

The High Level GVS was a deterministic, pseudo-random sum-of-sinusoids profile applied at 700 uA, which in pilot testing we found was disorienting and induced instability but caused little to no sensation on skin in order to blind the transition to sham between learning and aftereffect trials. Both GVS cues were zero-mean and generated by a current controlled stimulator (Soterix Medical Inc., Model 0810) which was customized to include the nGVS 0–30 Hz profile.

### Functional Mobility Task

**FIGURE 1:**
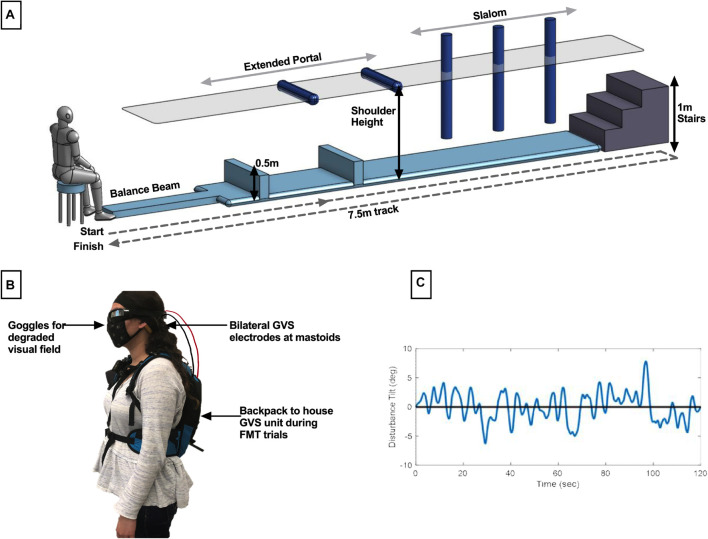
Description of methods. **(A)** The modified functional mobility task consisted of a series of obstacles placed along the length of an inflated air track. Hurdles to be ducked under were adjusted to shoulder height, and slaloms were adjusted to be shoulder width. **(B)** Member of the research team demonstrating electrode placement. For all subjects, bilateral GVS electrodes were placed at the mastoids. In the functional mobility task only, subjects wore goggles for the creation of a degraded visual field. To transport the GVS device with the subject through the course, it was placed in a low-profile backpack. **(C)** Sample computer-generated, pseudo-random, zero-mean sum-of-sines roll tilt motion profile. Subjects assigned to the manual control task were instructed to use a joystick to null this motion, with the goal of keeping their chair upright.

Subjects assigned to the functional mobility task (FMT) completed timed trials in an obstacle avoidance course (Figure 1A). This course is modeled after a similar course used at NASA’s Johnson Space Center to assess functional mobility of spaceflight participants after their flight experience (Mulavara et al., 2010) and which we have used recently (Dixon and Clark, 2020). The course consists of a series of obstacles that require balance, spatial awareness, and motor control for successful completion. Our course had a 7.5 m long linear design comprised of 1.5 m of foam balance beam followed by a 6 m long inflated air track for reduced proprioceptive cues, ending with a three-step stair climb. Hurdle obstacles had to be stepped over or ducked under, with the latter adjusted to each subject’s shoulder-height. Slalom hurdles were also adjustable in width spacing such that each subject could stand shoulder width in between each slalom.

Subjects were informed of the procedures for completing a trial through the course, where a trial is defined as starting seated at the beginning of the course, proceeding through each obstacle and up the stairs, turning around and proceeding through the obstacles in reverse order, and ending seated. Subjects were also informed that there were time penalties for incorrectly moving through the obstacles. Penalties included stepping off the balance beam, body contact with a hurdle in the extended portal or slalom, or using the handrails on the stairs to stabilize. Stepping off the air track at any point during the trial also resulted in a penalty. Subjects were informed that each occurrence of a penalty would add 5 s to their trial time. In data analysis, we found 2 s per incident to be a more suited penalty relative to the total average trial completion length. Subjects were given one practice trial, prior to the learning phase, to move through the course at their own pace without treatment to ensure they were familiar with the procedures and penalties. They were then instructed that their goal was to complete each trial as quickly as possible while minimizing penalties. The GVS unit was securely placed in a low-profile running backpack to move with subjects through the course (all subjects wore this throughout testing, regardless of whether it was a sham trial). The electrode cables were fed through an opening in the backpack and electrodes were held securely against the mastoids by an elastic headband as described above. Subjects also wore goggles to create a degraded visual field in order to reduce reliance on visual cues for completion of the course. The goggles were mirrored swim goggles with lower peripheral vision blockers (Figure 1B).

Subjects completed 12 FMT learning trials with GVS treatment and 8 aftereffects trials with sham GVS treatment. To reduce physical fatigue, subjects were given a 60 s break between each trial to rest. In order to blind the transition to sham, subjects were given a 90 s break instead of a standard 60 s break after every fourth trial, during which the operator would open the running backpack and check the GVS unit. On the third such break (after the 12th trial), the operator turned the GVS unit off without informing the subject.

### Manual Control Task

Subjects assigned to the manual control task completed a roll tilt nulling task using a custom human-rated motion device [the Tilt Translation Sled (TTS), without the translation axis activated]. Subjects were secured with a 5-point harness and head restraint. Manual control performance was assessed in the dark with auditory white noise playing through subject headphones to minimize non-vestibular cues. Subjects were instructed that they would experience a series of randomized head-centered disturbance tilts in the roll axis. As they experienced these tilts, their task was to use their joystick to null perceived tilt by executing opposing joystick inputs (i.e., to keep their chair as close to upright as possible throughout the 120 s trial). Joystick deflections were measured with a potentiometer, averaged over 1 s, and commanded chair roll tilt with a proportional gain (*K* = 2.5° of chair roll tilt per 1° of smoothed joystick deflection). Roll tilt disturbances were computer-generated, pseudo-random, zero-mean sum-of-sines profiles (Figure 1C). The generated profile was inverted by sign, reversed in time, or both inverted and reversed to create four randomized profiles comprised of the same 120 s of motion but preventing pattern recognition from the subject throughout trial completion. The first and last 5 s of each profile was scaled so that the chair disturbance began and ended at upright. These scaled portions were excluded from analysis. Maximum roll tilt disturbance was ±7.8°. Maximum joystick command was ±9° of chair tilt to allow for overcorrection.

Subjects completed 8 learning trials with their assigned GVS treatment followed by 5 aftereffects trials with sham GVS treatment. This distribution of trials was selected to match the approximate 45-min testing duration of the functional mobility task design. Subjects were blind to the transition between learning and aftereffects trials in a similar manner as for the functional mobility assessment. Joystick commands, as well as actual chair tilt, were recorded at 50 Hz. Performance was characterized by root mean square error (RMSE) of the chair nulled tilt angle, such that smaller values correspond to better performance.

### Direction Recognition Thresholds

Direction recognition thresholds were tested utilizing the TTS in roll tilt configuration. DR thresholds were only measured for manual control subjects, due to the relatively equivalent motion experience between the nulling task and roll-axis direction recognition. Roll-axis thresholds were not expected to be an effective prediction of inter-subject variability of performance on the multi-axis, multi-sensory FMT.

Subjects were seated and secured with a 5-point harness as well as a custom head restraint to keep the head en bloc with the body. Direction recognition thresholds were performed in the dark with auditory white noise to reduce non-vestibular cues. Operator commands were communicated through subject headphones. Roll tilt motions began at upright and consisted of a single-cycle sinusoid in angular acceleration, as is typically used in previous studies (Bermúdez Rey et al., 2016; Lim et al., 2017; Suri and Clark, 2020). The tilt profile was performed at 0.5 Hz frequency (2 s tilt duration). This tilt duration requires integration of otolith and semicircular canal cues (Lim et al., 2017; Suri and Clark, 2020). Subjects were tilted in the dark, and once the motion was complete while still tilted, were asked to verbally indicate which direction they perceived to be tilted, as well as their confidence in their answer. Confidence reports were provided in 5% increments, with 50% being a complete guess and 100% being complete certainty, however, the research on confidence adjusted thresholds is still ongoing thus are not further considered here. On each trial, tilt direction (i.e., left or right) was randomized, and tilt magnitude (in degrees) was adjusted using a 3 down 1 up staircase beginning at 6 degrees. Subjects completed 100 trials in order to produce a reliable threshold estimate (coefficient of variation of the estimate was theoretically ∼0.18) (Karmali et al., 2016). Subject responses were fit with a cumulative Gaussian psychometric curve (Merfeld, 2011). To accommodate the adaptive staircase, a bias-reduced generalized linear model (BRGLM) fit was performed with a probit link function (Chaudhuri and Merfeld, 2013), as is standard practice (Bermúdez Rey et al., 2016; Suri and Clark, 2020). The psychometric curve fit produces two parameters: μ – the vestibular bias (i.e., the stimulus at which the subject is equally likely to response “left” vs. “right,” and σ – which is commonly defined as the 1-sigma threshold (Merfeld, 2011), we adopt here. Psychometric curve fits to produce roll tilt thresholds were performed in MATLAB. Thresholds were log-transformed for normality (Suri and Clark, 2020).

### Data Analysis

**FIGURE 2:**
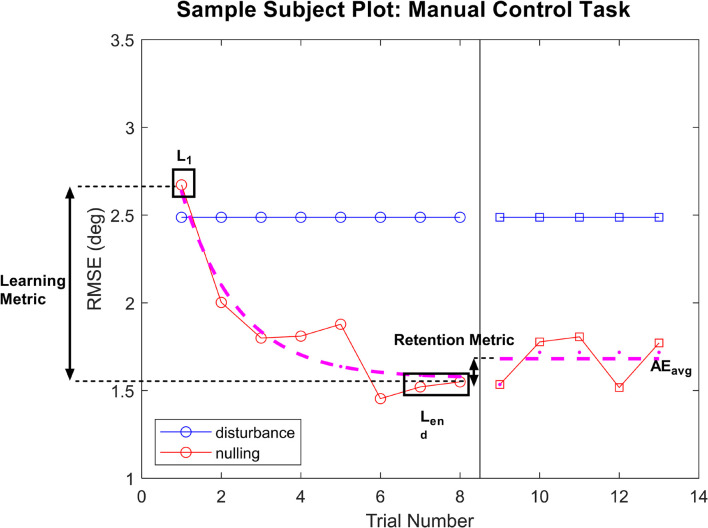
Sample plot of performance for a subject in the manual control task. For both tasks, performance on each trial was plotted for the learning phase and the aftereffects phase. An exponential decay was fit to the learning phase, with L_1_ and L_end_ values pulled from the model fits with *R*^2^ > 0.45. For subject whose learning was not well captured by the model fit (*R*^2^ < 0.45), raw performance on trials 1 or the average of trials 7 and 8 were used for L_1_ and L_en__d_, respectively. Average performance across the aftereffects phase was used as AE_avg_ in calculating the retention metric.

For each performance task, we fit a piecewise model as a function of trial number, in order to capture the learning and aftereffect responses. An example is provided of a subject in the manual control task in Figure 2.

During the learning phase, an exponential decay was fit to capture the improvement in performance as trials progressed. In particular, we calculated the model fit corresponding to initial performance and final learning phase performance [L(1) and L_e__nd_, respectively], where L_end_ averaged performance in the final two learning phase trials [L(7) and L(8) for manual control, L(11) and L(12) for FMT], and L(1) was taken as an independent measure of initial performance. The exponential model of learning is a well-used standard for measuring improvement of task performance with practice (Heathcote et al., 2000; Ritter et al., 2013). However, some subjects demonstrated learning that was not well captured by an exponential model. To quantify the appropriateness of the learning phase exponential model fit, *R*^2^ was calculated for each subject’s data. For individuals with a model fit *R*^2^ > 0.45, the exponential model was used to determine L(1) and L_e__nd_ parameters. For subjects whose performance was not well represented by the exponential decay model (*R*^2^ < 0.45), their raw trial performance was used to calculate L(1) and L_end_ parameters instead. Learning was defined as the difference in performance across the learning phase relative to the subject’s initial performance during this phase, using the following equation:


$$\text{Learning}\text{Metric}=\frac{L\left(1\right)-L_{end}}{L\left(1\right)}$$

A high learning metric score indicates that the subject had a large improvement of performance from their initial performance (L_1_) to their performance at the end of the learning phase (L_end_), indicating more learning. If the learning metric is zero, no change in performance occurs during this phase.

During the aftereffect phase, average performance across all trials within that phase was computed (AE_avg_) and compared to performance at the end of the learning phase (L_end_ as previously described). A retention metric was calculated, which corresponds to the average performance when GVS was removed relative to the subject’s performance at the end of the learning phase, and was calculated using the following equation:


$$\text{Retention}\text{Metric}=\frac{L_{end}-AE_{avg}}{L_{end}}$$

If the subject’s performance did not change from the end of the learning phase to the aftereffect phase (i.e., there was no change when the GVS was blinded turned off), the retention metric would be zero. If performance improved after GVS was turned off, the retention metric would be positive, while it would be negative if performance degraded (e.g., a subject lost some of the learning accomplished during GVS treatment).

## Results

### Functional Mobility Task

**FIGURE 3:**
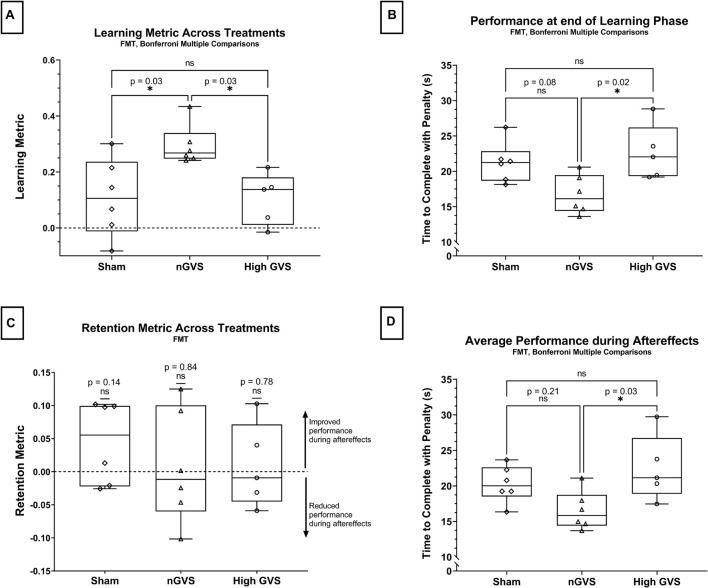
FMT data analysis. The symbol * denotes significant comparisons at α = 0.05. **(A)** Comparison of learning metric across treatments. **(B)** Comparison of time to complete the course (with penalties) at the end of the learning phase (L_end_) across treatments. **(C)** Comparison of retention metric across treatments shows no significant change in performance between end of learning phase and average performance during aftereffects for any treatment. **(D)** Average time to complete the course (with penalties) during aftereffects (AE_avg_) across treatment groups.

In the functional mobility task, a one-way ANOVA showed a significant difference in learning metric across GVS treatment groups [*F*(2,14) = 6.09, *p* = 0.013]. Pairwise comparisons with a Bonferroni adjustments demonstrated that subjects receiving nGVS treatment showed a significant increase in learning of 171% compared to sham [*t*(14) = 3.03, *p* = 0.027], as well as a significant increase in learning of 184% compared to the high GVS condition [*t*(14) = 2.97, *p* = 0.031]. No significant difference in task learning was observed between sham and high GVS treatments [High GVS learning 5% lower than sham, *t*(14) = 0.08, *p* > 0.99 with Bonferroni adjustment]. These results can be seen in Figure 3A. Due to the low sample size in this investigation, non-parametric analysis was also performed. Kruskal–Wallis testing demonstrated global significance between learning metric across GVS treatment groups [H(2) = 8.542, *p* = 0.007]. *Post hoc* testing using Dunn’s multiple comparisons showed a significantly higher learning metric in nGVS subject compared to sham (*p* = 0.04) as well as high GVS subjects (*p* = 0.03), but no significant difference in learning metric between sham and high GVS subjects (*p* > 0.99) (matching the parametric test conclusions in each case). For remaining analysis in this investigation, tests of normality and appropriate assumptions were used to select parametric vs. non-parametric analysis. Unless reported otherwise, results of non-parametric testing matched those of parametric testing.

Next, we considered the raw performance at the end of the learning phase (L_end_), in which a one-way ANOVA showed a significant difference in performance across treatment groups [*F*(2,14) = 5.46, *p* = 0.018]. This analysis further demonstrates that subjects receiving nGVS treatment finished the learning phase with marked improvement compared to other treatment groups. nGVS subjects showed significantly faster trial completion than high GVS subjects, with a mean difference in trial time 5.91 s faster [*t*(14) = 2.49, *p* = 0.024], and marginal significance compared to sham subjects with a mean difference 4.53 s faster [*t*(14) = 3.096, *p* = 0.08]. No significant difference in performance at the end of the learning phase was seen between sham and high GVS subjects [*t*(14) = 0.723, *p* > 0.99] (Figure 3B).

We tested the retention metric across treatments against a theoretical mean = 0, which would indicate no change in performance between learning and aftereffects phases. Two-tailed *t*-tests showed no significant difference from zero for sham [*t*(5) = 1.64, *p* = 0.14], nGVS [*t*(5) = 0.219, *p* = 0.84], or high GVS [*t*(4) = 0.302, *p* = 0.78], demonstrating that change in performance during the learning phase was retained into the aftereffects phase when nGVS and high GVS treatments were transitioned to sham. These results also demonstrate that high GVS subjects showed no significant improvement in performance during the aftereffects phase as might be expected when removing the destabilizing treatment (Figure 3C).

Finally, analysis of averaged performance throughout the aftereffects phase was used to examine how subject performance differed beyond the learning phase. This analysis was performed to determine if nGVS subjects remained the fastest performance group even once treatment was removed. One-way ANOVA showed a significant difference between average aftereffects performance across treatment groups [*F*(2,14) = 4.552, *p* = 0.03]. *Post hoc* comparisons with a Bonferroni adjustment show that nGVS subjects remain significantly faster in trial completion than high GVS subjects, with a mean difference in trial time 5.97 s faster [*t*(14) = 2.96, *p* = 0.031], but no significant difference compared to sham subjects [mean difference 3.74 s faster in nGVS subjects, *t*(14) = 1.943, *p* = 0.22]. No significant difference in trial completion time was seen between sham and high GVS subjects [mean difference 2.23 s faster in sham subjects, *t*(14) = 1.10, *p* = 0.86] (Figure 3D).

### Manual Control Task

**FIGURE 4:**
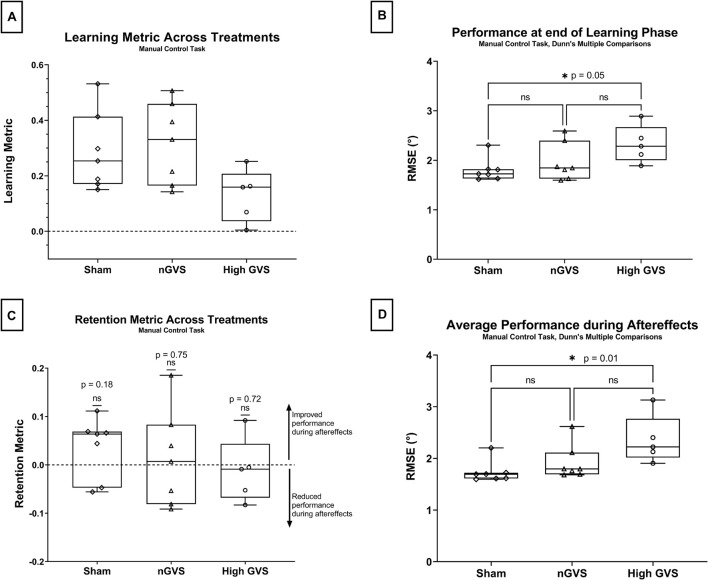
Manual control data analysis. The symbol * denotes significant comparisons at α = 0.05. **(A)** Comparison of learning metric across treatments. No global significance between treatments. **(B)** Comparison of nulling performance at the end of the learning phase (L_end_) across treatments. **(C)** Comparison of retention metric across treatments shows no significant change in performance between end of learning phase and average performance during aftereffects for any treatment. **(D)** Average nulling performance during aftereffects (AE_avg_) across treatment groups.

In the manual control task, a one-way ANOVA approached but did not reach a significant difference in learning metric across GVS treatment groups [*F*(2,16) = 3.19, *p* = 0.07] (Figure 4A). nGVS subjects showed an average learning metric 10% higher than subjects receiving sham and 59% higher than subjects receiving high GVS treatment.

Performance at the end of the learning phase (L_end_) failed Shapiro–Wilk tests of normality for sham (*p* = 0.014) subjects, thus non-parametric test were used for hypothesis testing. Kruskal–Wallis testing showed a significant difference in performance at L_end_ across treatment groups [H(2) = 6.01, *p* = 0.04]. A pairwise *post hoc* Dunn test was significant for sham vs. high GVS (*p* = 0.046 but not sham vs. nGVS (*p* > 0.99) or nGVS vs. high GVS (*p* = 0.27) as seen in Figure 4B.

The retention metrics across treatments for this task were also tested against a theoretical mean = 0, as described above. Two-tailed *t*-tests showed no significant difference from zero for sham [*t*(6) = 1.503, *p* = 0.18], nGVS [*t*(6) = 0.337, *p* = 0.75], or high GVS [*t*(4) = 0.390, *p* = 0.72], demonstrating that change in performance during the learning phase was retained into the aftereffects phase when nGVS and high GVS treatments were transitioned to sham. These results also demonstrate that high GVS subjects in the manual control task also showed no significant improvement in performance during the aftereffects phase, again as might be expected (Figure 4C).

Average performance throughout the aftereffects phase (AE_avg_) also failed Shapiro–Wilk tests of normality for sham (*p* = 0.001) and nGVS treatments (*p* = 0.011), thus non-parametric tests were used for hypothesis testing. A Kruskal–Wallis test showed a significant difference in performance at L_end_ across treatment groups [H(2) = 8.49, *p* = 0.008]. A pairwise *post hoc* Dunn test was significant for sham vs. high GVS (*p* = 0.01) but not sham vs. nGVS (*p* = 0.55) or nGVS vs. high GVS (*p* = 0.27) as seen in Figure 4D.

### Roll Tilt Direction Recognition Thresholds for Manual Control Subjects

**FIGURE 5:**
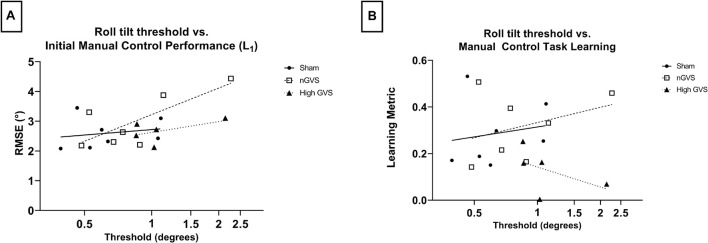
Correlating DR threshold and task performance for manual control subjects. **(A)** While all subjects show a positive correlation between DR threshold and initial task performance, this relationship is only significant for nGVS subjects (*p* = 0.05). **(B)** No significant correlation between DR threshold and manual control task learning across any treatment group.

Simple linear regression was performed to investigate the relationship between roll tilt direction recognition thresholds and manual control performance measured by both initial performance (L_1_) and learning metric. DR thresholds were log transformed for normality. In examining L_1_ performance, linear best fit lines show a positive correlation between performance and threshold across all treatments. However, only nGVS subjects showed a significantly non-zero slope [*F*(1,5) = 6.739, *p* = 0.05, *R*^2^ = 0.57]. Neither sham nor high GVS subjects showed significant slopes (*R*^2^ = 0.04 and 0.28, respectively) (Figure 5A). In examining the relationship between threshold and learning during the manual control task as assessed by the learning metric, no treatments showed a significant slope. However, sham and nGVS subjects showed a best fit line with a positive slope (*R*^2^ = 0.03 and 0.12, respectively) while high GVS subjects showed a best fit line with a negative slope (*R*^2^ = −0.49) (Figure 5B). Due to low sample size, non-parametric analysis of correlation through Spearman Rank was also performed. No significant correlation between DR threshold and L_1_ was seen for sham (Spearman *r* = 0.36, *p* = 0.44), nGVS (*r* = 0.68, *p* = 0.11) or high GVS subjects (*r* = 0.50, *p* = 0.45). Similarly, no significant correlation between DR threshold and learning metric was seen for sham (*r* = 0.29, *p* = 0.56), nGVS (*r* = 0.29, *p* = 0.56), or high GVS subjects (*r* = −0.50, *p* = 0.45).

## Discussion

### How Does Noisy Galvanic Vestibular Stimulation Treatment Affect Sensorimotor Learning in Functional Mobility and Manual Control Tasks?

Analysis of functional mobility data supports that nGVS treatment improved performance across the learning phase, with a statistically significant higher learning metric compared to sham. This improvement in performance was retained even once GVS treatment was removed, as indicated by retention metrics for the nGVS subject group that were not significantly different than zero (retention metric = 0 indicates no change in performance between the end of the learning phase and the aftereffect phase). Analysis of manual control nulling task data showed no significant difference in learning metric between GVS groups, and no significant difference in performance at the end of the learning phase or average performance during aftereffects between sham and nGVS subjects in this task. As an initial investigation of the effect of GVS on learning of sensorimotor tasks, we acknowledge the low sample size in this study. Effort was taken to interpret results with caution and use non-parametric analysis when appropriate.

Learning metric differences between manual control and FMT performance suggests that nGVS provides benefit to learning for tasks like FMT which involve multisensory integration, body coordination, and strategy. Previous studies of GVS support enhancement in functions essential in functional mobility task success, such as gait (Iwasaki et al., 2018), locomotor stability (Mulavara et al., 2015), and dynamic balance (Stefani et al., 2020). A 2019 study showed evidence that nGVS enhances spatial memory, an essential part of obstacle avoidance and overall success in course completion (Hilliard et al., 2019). Further, another study found improved visual perceptual thresholds during nGVS application, suggesting cross-modal stochastic resonance (Voros et al., 2021). In contrast, the manual control task is mostly a vestibular sensation and motor reaction task. It is possible that nGVS is unable to enhance learning of such a sensorimotor task that is predominantly vestibular perception related. The small sample size in this study may have prevented a measurable effect of nGVS on learning of the manual control task, however, the data do not trend toward a positive relationship between this treatment and improved performance.

Alternatively, it is worth noting that in a previous study that found nGVS to improve roll tilt direction recognition thresholds (Galvan-Garza et al., 2018), the investigators used subject-specific optimal stimulation levels identified from either 200, 300, 500, or 700 uA. In this study the group optimal level was 300 uA, which motivated our use of that single level to investigate sensorimotor learning with nGVS. Many previous studies have calculated subject-specific optimal stimulation levels, frequently using postural sway tasks (Fujimoto et al., 2016; Iwasaki et al., 2018; Keywan et al., 2018, 2019, 2020). As such, differences in learning metric across nGVS subject could be a result of the use of a single nGVS current level of 300 uA for all nGVS subjects. It may be that 300 uA happened to be optimal or near optimal for some subjects, and less so for other subjects, such that they produced less clear improvements with nGVS in our study, but might have if we were able to optimized the nGVS treatment for them. Since learning was a primary metric in this study, it was not possible to perform repeated measures of performance with varying stimulation levels in order to determine optimal stimulation, as subjects would learn during this time and that learning would not be captured. In order to determine subject-specific optimal levels, we would need to test subjects on a separate task. Doing so assumes that optimal GVS stimulation level for once task, such as a postural sway assessment, is also a subject’s optimal for another task, in this case functional mobility or manual control. We did not feel confident making that assumption, thus chose 300 uA as the treatment level for all nGVS subjects. Future work to explore how optimal GVS levels differ across a multitude of tests such as postural sway, gait or dynamic balance, functional mobility, manual control, or direction recognition thresholds may support future selection of subject-specific optimal stimulation levels, particularly for learning of sensorimotor tasks.

Improvement in performance during learning was retained even once GVS treatment had been removed for both tasks. This result suggests that application of nGVS treatment during active task learning could provide sustained operational benefits. Previous studies have found that passive GVS treatment before task completion does not show evidence of aftereffects (Keywan et al., 2020). This difference in results suggests that sustained improvement is only achieved when nGVS is applied *during active learning of a task*. However, retention was only measured for approximately 15 min of aftereffects performance in both tasks. Long-duration retention of improvement cannot be concluded from the results of this study. Future work should explore retesting subject performance hours or even days after learning with active stimulation was completed in order to assess long-term sustained retention of improvement. Nonetheless, it is highly promising that the benefits of nGVS in enhancing functional mobility learning, as compared to sham, are sustained even after the nGVS is turned off.

### How Does High Galvanic Vestibular Stimulation Treatment Affect Sensorimotor Learning in Functional Mobility and Manual Control Tasks?

High GVS treatment caused some impairment of learning, however, this impairment was not significantly different from sham for either functional mobility or manual control. Removal of treatment in aftereffects did not prompt increased learning for either task, as might be expected when deactivating the destabilizing treatment.

The original intention of the high-level stimulation was to create a disruptive and difficult environment for learning. If learning was conducted in a disruptive environment, it was hypothesized that removal of that disruption and easing of the task environment may prompt improved aftereffects performance. This method of learning can be likened to resistance training, where removing additional weights or resistance that were used in training prompts increased performance in an “easier” environment. Previous studies have used high levels of GVS to create a disruptive balance environment (MacDougall et al., 2006; Sloot et al., 2011).

High GVS treatment as used in this study may not have caused improved learning when removed due to the stimulation profile selected not providing a sufficiently disruptive learning environment. Sloot et al. (2011) used pseudo-random sum of sinusoids with a maximum amplitude of 2.2 mA of current to create balance impairment. MacDougall et al. (2006) used an even higher profile to create disruption, with stimulation up to 5 mA. An additional study which found high level GVS to disrupt body ownership and “self-advantage” also used stimulation levels with a maximum of 5 mA (Hoover and Harris, 2015). However, stimulation in this study was selected to be disruptive while minimizing skin surface sensation in order to keep skin sensitivity blinded through the transition between learning and aftereffects, when all subjects were transitioned to sham. Pilot testing found that current levels about ∼750 uA were frequently detectable via skin surface sensations, thus the High GVS treatment was set at a maximum intensity of 700 uA. High level subjects tested in this study showed no significant improvement in performance during the learning phase for either functional mobility or manual control, demonstrating that the 700 uA stimulation level was indeed somewhat disruptive to learning. However, this level may not have been disruptive enough to instigate aftereffects learning as originally hypothesized.

### Are Roll Tilt Direction Recognition Thresholds an Indicator of Inter-Subject Variability of Performance in Manual Control Tasks?

Simple linear regression found a significant and positive correlation between roll tilt direction recognition threshold and initial manual control task performance for nGVS subjects, but not sham or high GVS subjects. No significant correlation was found between an individual’s threshold and learning metric for any treatment. However, only high GVS subjects showed a negative correlation between threshold and learning. This result may indicate that task learning is least disrupted by high GVS treatment for subjects with lower baseline roll tilt thresholds. While not significant, the magnitude of impact of GVS treatment on learning trended higher for subjects with a higher baseline threshold, while subjects with lower baseline thresholds experienced learning more similar to sham. It should be noted that this analysis was performed only for subjects assigned to the manual control task, resulting in a low n and a reduced continuum of possible baseline thresholds for each treatment group. Preliminary trends seen here may pose an area of exploration for a future, higher power study focused on exploring inter-subject variability in sensorimotor task performance. Our study was not powered for or designed to explore sex differences in response to GVS. However, previous research has found no sex differences in sensorimotor adaptations to gravity transitions, nor in vestibular perceptual thresholds (Reschke et al., 2014; Bermúdez Rey et al., 2016). Based on this literature, we did not explore sex as a variable in our analysis.

### Operational Context

Based on the results reported here, nGVS may be an effective tool to improve the learning of complex sensorimotor tasks like the functional mobility task. This has strong operational applications, particularly for astronauts. Post-flight sensorimotor impairment poses risks for mission essential task completion in orbit as well as safety when returning to earth (Bloomberg and Mulavara, 2003; Bloomberg, 2015; Bloomberg and Madansingh, 2015; Clark, 2019). Perhaps more importantly, maladaptation in long duration spaceflight poses significant risks to safety and successful task completion for mission essential surface operations in Moon or Mars missions. When astronauts arrive to a new planetary surface, they will have to “relearn” performance of tasks in their new gravity field and environment, especially when adapting to the weight and mobility challenges of a spacesuit. nGVS may be useful to improve task performance through application during active performance of tasks, as demonstrated in this study. It may also enhance learning of tasks as astronauts adapt to their new environment. Even if application is only done for initial periods of task learning and completion, the results of this study demonstrate that improvement in performance is retained even when nGVS treatment has been removed.

Beyond spaceflight applications, nGVS has the potential to benefit any individual for which functional mobility is an essential part of their work, such as firefighters, high performance athletes, or soldiers. While we only studied healthy participants, the benefits of nGVS also have the potential to aid elderly people or patients recovering from a condition that prevented locomotion. The findings of this study supporting the sustained benefit of nGVS when applied during active task learning can be used to implement GVS as a tool for all of these groups. In this preliminary study of sensorimotor learning, we acknowledge small sample size limitations on our findings. We believe that the results of this study justify further exploration into the effect of GVS on sensorimotor learning.

## Data Availability Statement

The raw data supporting the conclusions of this article will be made available by the authors, without undue reservation.

## Ethics Statement

The studies involving human participants were reviewed and approved by the CU Boulder Institutional Review Board (IRB Protocol Number 20-0097). The patients/participants provided their written informed consent to participate in this study.

## Author Contributions

EP carried out all subject testing, collected the data, wrote the manuscript with support from TC and RG-G analyzed the data, and interpreted the results with the support of TC. RG-G and TC conceived of the experiment and designed it with support from EP. All authors contributed to the article and approved the submitted version.

## Conflict of Interest

RG-G was employed by company Lockheed Martin Advanced Technology Laboratories. This study received funding from Lockheed Martin Corporation. The funder had the following involvement with the study: study design, review of data, decision to publish, and review of the manuscript.

## Publisher’s Note

All claims expressed in this article are solely those of the authors and do not necessarily represent those of their affiliated organizations, or those of the publisher, the editors and the reviewers. Any product that may be evaluated in this article, or claim that may be made by its manufacturer, is not guaranteed or endorsed by the publisher.

## References

[B1] Aihara T. Kitajo K. Nozaki D. Yamamoto Y. (2010). How does stochastic resonance work within the human brain? – Psychophysics of internal and external noise. *Chem. Phys.* 375 616–624. 10.1016/j.chemphys.2010.04.027

[B2] Bermúdez Rey M. C. Clark T. K. Wang W. Leeder T. Bian Y. Merfeld D. M. (2016). Vestibular perceptual thresholds increase above the age of 40. *Front. Neurol.* 7:162. 10.3389/fneur.2016.00162 27752252 PMC5046616

[B3] Bloomberg J. J. Madansingh S. (2015). Understanding the effects of spaceflight on head–trunk coordination during walking and obstacle avoidance. *Acta Astronaut.* 115 165–172. 10.1016/j.actaastro.2015.05.022

[B4] Bloomberg J. J. Mulavara A. P. (2003). Changes in walking strategies after spaceflight. *IEEE Eng. Med. Biol. Mag.* 22 58–62. 10.1109/MEMB.2003.1195697 12733460

[B5] Bloomberg J. J. R. (2015). *Risk of Impaired Control of Spacecraft/Associated Systems and Decreased Mobility Due to Vestibular/Sensorimotor Alterations Associated with Space flight.* Available online at: https://ntrs.nasa.gov/search.jsp?R=20150018603 (accessed April 7, 2020).

[B6] Chaudhuri S. E. Merfeld D. M. (2013). Signal detection theory and vestibular perception: III. Estimating unbiased fit parameters for psychometric functions. *Exp. Brain Res.* 225 133–146. 10.1007/s00221-012-3354-7 23250442 PMC3570703

[B7] Clark T. K. (2019). “Effects of spaceflight on the vestibular system,” in *Handbook of Space Pharmaceuticals*, eds Pathak Y. Araújo dos Santos M. Zea L. (Cham: Springer International Publishing), 1–39. 10.1007/978-3-319-50909-9_2-1

[B8] Dixon J. B. Clark T. K. (2020). Sensorimotor impairment from a new analog of spaceflight-altered neurovestibular cues. *J. Neurophysiol.* 123 209–223. 10.1152/jn.00156.2019 31747329

[B9] Ferrè E. R. Vagnoni E. Haggard P. (2013c). Vestibular contributions to bodily awareness. *Neuropsychologia* 51 1445–1452. 10.1016/j.neuropsychologia.2013.04.006 23624312

[B10] Ferrè E. R. Day B. L. Bottini G. Haggard P. (2013a). How the vestibular system interacts with somatosensory perception: a sham-controlled study with galvanic vestibular stimulation. *Neurosci. Lett.* 550 35–40. 10.1016/j.neulet.2013.06.046 23827220 PMC3988931

[B11] Ferrè E. R. Longo M. Fiori F. Haggard P. (2013b). Vestibular modulation of spatial perception. *Front. Hum. Neurosci.* 7:660. 10.3389/fnhum.2013.00660 24133440 PMC3794195

[B12] Ferrè E. R. Lopez C. Haggard P. (2014). Anchoring the self to the body: vestibular contribution to the sense of self. *Psychol. Sci.* 25 2106–2108. 10.1177/0956797614547917 25210013

[B13] Fujimoto C. Yamamoto Y. Kamogashira T. Kinoshita M. Egami N. Uemura Y. (2016). Noisy galvanic vestibular stimulation induces a sustained improvement in body balance in elderly adults. *Sci. Rep.* 6 1–8. 10.1038/srep37575 27869225 PMC5116631

[B14] Galvan-Garza R. (2016). *Enhancement of Perception with the Application of Stochastic Vestibular Stimulation.* Available online at: https://dspace.mit.edu/handle/1721.1/107550 (accessed March 10, 2021).

[B15] Galvan-Garza R. C. Clark T. K. Mulavara A. P. Oman C. M. (2018). Exhibition of stochastic resonance in vestibular tilt motion perception. *Brain Stimul.* 11 716–722. 10.1016/j.brs.2018.03.017 29656906

[B16] Goel R. Kofman I. Jeevarajan J. De Dios Y. Cohen H. S. Bloomberg J. J. (2015). Using low levels of stochastic vestibular stimulation to improve balance function. *PLoS One* 10:e0136335. 10.1371/journal.pone.0136335 26295807 PMC4546608

[B17] Heathcote A. Brown S. Mewhort D. J. K. (2000). The power law repealed: the case for an exponential law of practice. *Psychon. Bull. Rev.* 7 185–207. 10.3758/BF03212979 10909131

[B18] Hilliard D. Passow S. Thurm F. Schuck N. W. Garthe A. Kempermann G. (2019). Noisy galvanic vestibular stimulation modulates spatial memory in young healthy adults. *Sci. Rep.* 9:9310. 10.1038/s41598-019-45757-0 31249334 PMC6597709

[B19] Hoover A. E. N. Harris L. R. (2015). Disrupting vestibular activity disrupts body ownership. *Multisens. Res.* 28 581–590. 10.1163/22134808-00002472 26595957

[B20] Inukai Y. Masaki M. Otsuru N. Saito K. Miyaguchi S. Kojima S. (2018a). Effect of noisy galvanic vestibular stimulation in community-dwelling elderly people: a randomised controlled trial. *J. NeuroEng. Rehabil.* 15:63. 10.1186/s12984-018-0407-6 29970144 PMC6029379

[B21] Inukai Y. Otsuru N. Masaki M. Saito K. Miyaguchi S. Kojima S. (2018b). Effect of noisy galvanic vestibular stimulation on center of pressure sway of static standing posture. *Brain Stimul.* 11 85–93. 10.1016/j.brs.2017.10.007 29079459

[B22] Iwasaki S. Fujimoto C. Egami N. Kinoshita M. Togo F. Yamamoto Y. (2018). Noisy vestibular stimulation increases gait speed in normals and in bilateral vestibulopathy. *Brain Stimul.* 11 709–715. 10.1016/j.brs.2018.03.005 29563049

[B23] Iwasaki S. Yamamoto Y. Togo F. Kinoshita M. Yoshifuji Y. Fujimoto C. (2014). Noisy vestibular stimulation improves body balance in bilateral vestibulopathy. *Neurology* 82 969–975. 10.1212/WNL.0000000000000215 24532279

[B24] Karmali F. Bermúdez Rey M. C. Clark T. K. Wang W. Merfeld D. M. (2017). Multivariate analyses of balance test performance, vestibular thresholds, and age. *Front. Neurol.* 8:578. 10.3389/fneur.2017.00578 29167656 PMC5682300

[B25] Karmali F. Chaudhuri S. E. Yi Y. Merfeld D. M. (2016). Determining thresholds using adaptive procedures and psychometric fits: evaluating efficiency using theory, simulations, and human experiments. *Exp. Brain Res.* 234 773–789. 10.1007/s00221-015-4501-8 26645306 PMC4831214

[B26] Karmali F. Goodworth A. D. Valko Y. Leeder T. Peterka R. J. Merfeld D. M. (2021). The role of vestibular cues in postural sway. *J. Neurophysiol.* 125 672–686. 10.1152/jn.00168.2020 33502934 PMC7948142

[B27] Keywan A. Badarna H. Jahn K. Wuehr M. (2020). No evidence for after-effects of noisy galvanic vestibular stimulation on motion perception. *Sci. Rep.* 10 1–7. 10.1038/s41598-020-59374-9 32054910 PMC7018946

[B28] Keywan A. Jahn K. Wuehr M. (2019). Noisy galvanic vestibular stimulation primarily affects otolith-mediated motion perception. *Neuroscience* 399 161–166. 10.1016/j.neuroscience.2018.12.031 30593921

[B29] Keywan A. Wuehr M. Pradhan C. Jahn K. (2018). Noisy galvanic stimulation improves roll-tilt vestibular perception in healthy subjects. *Front. Neurol.* 9:83. 10.3389/fneur.2018.00083 29545766 PMC5837962

[B30] Lim K. Karmali F. Nicoucar K. Merfeld D. M. (2017). Perceptual precision of passive body tilt is consistent with statistically optimal cue integration. *J. Neurophysiol.* 117 2037–2052. 10.1152/jn.00073.2016 28179477 PMC5434481

[B31] MacDougall H. G. Moore S. T. Curthoys I. S. Black F. O. (2006). Modeling postural instability with galvanic vestibular stimulation. *Exp. Brain Res.* 172 208–220. 10.1007/s00221-005-0329-y 16432695

[B32] McDonnell M. D. Abbott D. (2009). What is stochastic resonance? Definitions, misconceptions, debates, and its relevance to biology. *PLoS Comput. Biol.* 5:e1000348. 10.1371/journal.pcbi.1000348 19562010 PMC2660436

[B33] Merfeld D. M. (2011). Signal detection theory and vestibular thresholds: I. Basic theory and practical considerations. *Exp. Brain Res.* 210 389–405. 10.1007/s00221-011-2557-7 21359662 PMC3096492

[B34] Moore S. T. Dilda V. MacDougall H. G. (2011). Galvanic vestibular stimulation as an analogue of spatial disorientation after spaceflight. *Aviat. Space Environ. Med.* 82 535–542. 10.3357/ASEM.2942.2011 21614868

[B35] Moore S. T. MacDougall H. G. Peters B. T. Bloomberg J. J. Curthoys I. S. Cohen H. S. (2006). Modeling locomotor dysfunction following spaceflight with Galvanic vestibular stimulation. *Exp. Brain Res.* 174 647–659. 10.1007/s00221-006-0528-1 16763834

[B36] Moss F. Ward L. M. Sannita W. G. (2004). Stochastic resonance and sensory information processing: a tutorial and review of application. *Clin. Neurophysiol.* 115 267–281. 10.1016/j.clinph.2003.09.014 14744566

[B37] Mulavara A. P. Feiveson A. H. Fiedler J. Cohen H. Peters B. T. Miller C. (2010). Locomotor function after long-duration space flight: effects and motor learning during recovery. *Exp. Brain Res.* 202 649–659. 10.1007/s00221-010-2171-0 20135100

[B38] Mulavara A. P. Fiedler M. J. Kofman I. S. Wood S. J. Serrador J. M. Peters B. (2011). Improving balance function using vestibular stochastic resonance: optimizing stimulus characteristics. *Exp. Brain Res.* 210 303–312. 10.1007/s00221-011-2633-z 21442221

[B39] Mulavara A. P. Kofman I. S. De Dios Y. E. Miller C. Peters B. T. Goel R. (2015). Using low levels of stochastic vestibular stimulation to improve locomotor stability. *Front. Syst. Neurosci.* 9:117. 10.3389/fnsys.2015.00117 26347619 PMC4547107

[B40] Nooristani M. Maheu M. Houde M.-S. Bacon B.-A. Champoux F. (2019). Questioning the lasting effect of galvanic vestibular stimulation on postural control. *PLoS One* 14, e0224619. 10.1371/journal.pone.0224619 31697727 PMC6837330

[B41] Reason J. T. (1968). Relations between motion sickness susceptibility, the spiral after-effect and loudness estimation. *Br. J. Psychol.* 59 385–393. 10.1111/j.2044-8295.1968.tb01153.x 5719793

[B42] Reschke M. F. Clément G. (2018). Vestibular and sensorimotor dysfunction during space flight. *Curr. Pathobiol. Rep.* 6 177–183. 10.1007/s40139-018-0173-y

[B43] Reschke M. F. Cohen H. S. Cerisano J. M. Clayton J. A. Cromwell R. Danielson R. W. (2014). Effects of sex and gender on adaptation to space: neurosensory systems. *J. Womens Health* 23 959–962. 10.1089/jwh.2014.4908 25401941 PMC4236059

[B44] Ritter F. Baxter G. Kim J. W. Srinivasmurthy S. (2013). “Learning and retention,” in *The Oxford Handbook of Cognitive Engineering*, eds Lee J. D. Kirlik A. (Oxford: Oxford University Press), 125–142.

[B45] Rosenberg M. J. Galvan-Garza R. C. Clark T. K. Sherwood D. P. Young L. R. Karmali F. (2018). Human manual control precision depends on vestibular sensory precision and gravitational magnitude. *J. Neurophysiol.* 120 3187–3197. 10.1152/jn.00565.2018 30379610 PMC6442919

[B46] Sloot L. H. van Schooten K. S. Bruijn S. M. Kingma H. Pijnappels M. van Dieën J. H. (2011). Sensitivity of Local dynamic stability of over-ground walking to balance impairment due to galvanic vestibular stimulation. *Ann. Biomed. Eng.* 39 1563–1569. 10.1007/s10439-010-0240-y 21222163 PMC3071943

[B47] Stefani S. P. Serrador J. M. Breen P. P. Camp A. J. (2020). Impact of galvanic vestibular stimulation-induced stochastic resonance on the output of the vestibular system: a systematic review. *Brain Stimul.* 13 533–535. 10.1016/j.brs.2020.01.006 32289671

[B48] Suri K. Clark T. K. (2020). Human vestibular perceptual thresholds for pitch tilt are slightly worse than for roll tilt across a range of frequencies. *Exp. Brain Res.* 238 1499–1509. 10.1007/s00221-020-05830-x 32444940

[B49] Utz K. S. Dimova V. Oppenländer K. Kerkhoff G. (2010). Electrified minds: transcranial direct current stimulation (tDCS) and galvanic vestibular stimulation (GVS) as methods of non-invasive brain stimulation in neuropsychology–a review of current data and future implications. *Neuropsychologia* 48 2789–2810. 10.1016/j.neuropsychologia.2010.06.002 20542047

[B50] Voros J. L. Sherman S. O. Rise R. Kryuchkov A. Stine P. Anderson A. P. (2021). Galvanic vestibular stimulation produces cross-modal improvements in visual thresholds. *Front. Neurosci.* 15:640984. 10.3389/fnins.2021.640984 33867923 PMC8044370

