# Supplementary figures and images for: The Effect of Noisy Galvanic Vestibular Stimulation on Learning of Functional Mobility and Manual Control Nulling Sensorimotor Tasks
Source: Front Hum Neurosci. 2021 Nov 3;15:756674. doi: 10.3389/fnhum.2021.756674 (PMC8595260; doi:10.3389/fnhum.2021.756674)

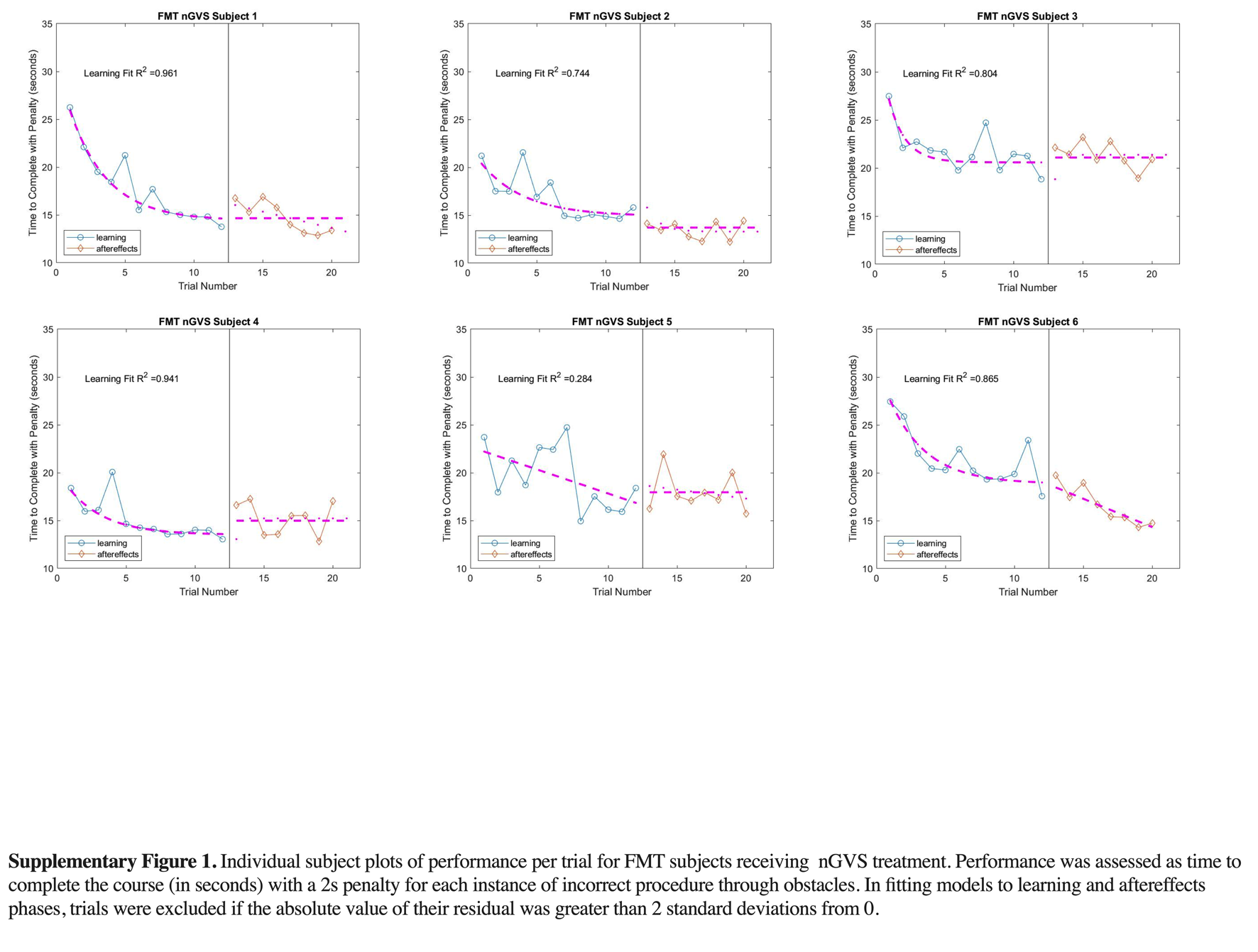

Supplement: Supplementary file 1 [file Image_1.TIFF]

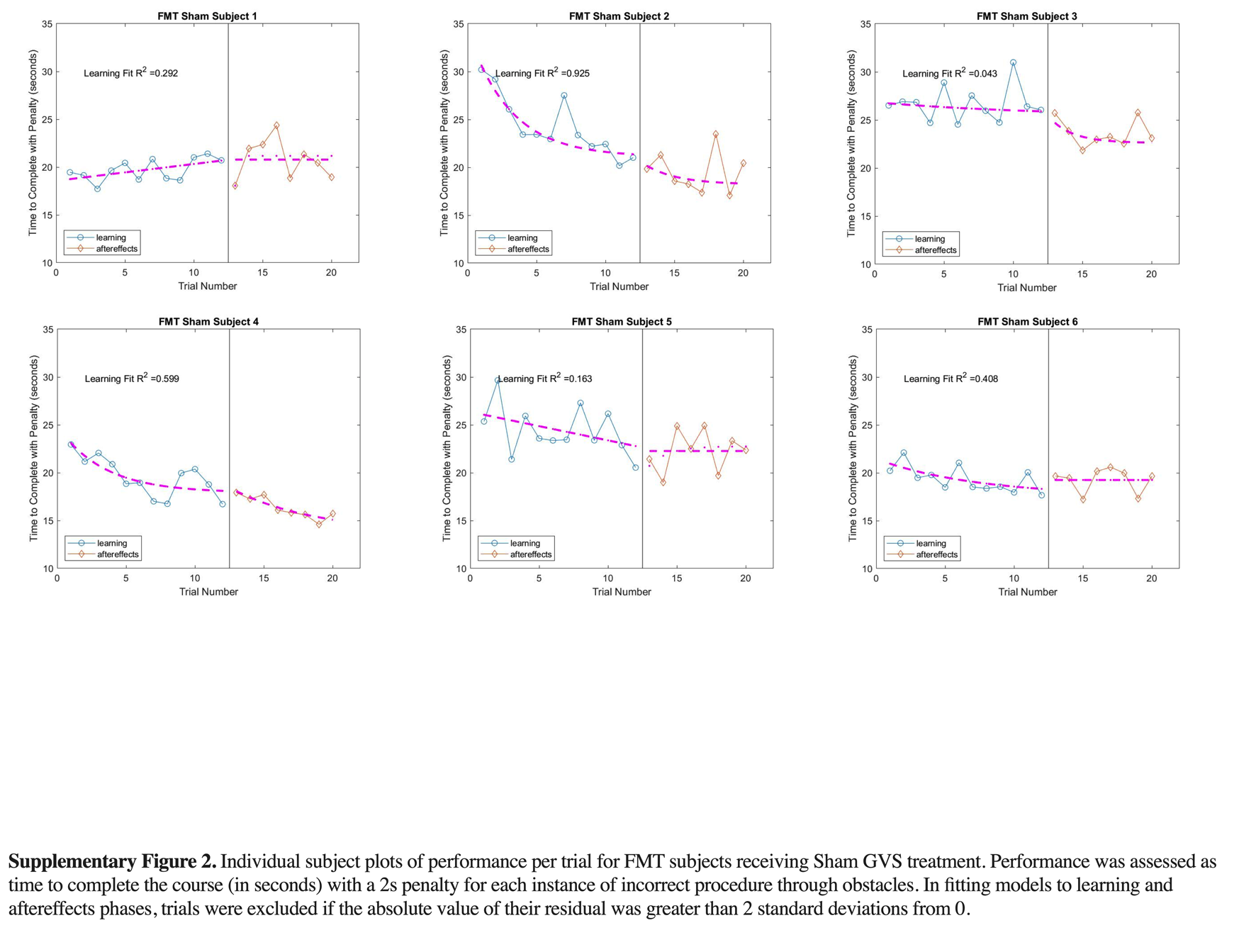

Supplement: Supplementary file 2 [file Image_2.TIFF]

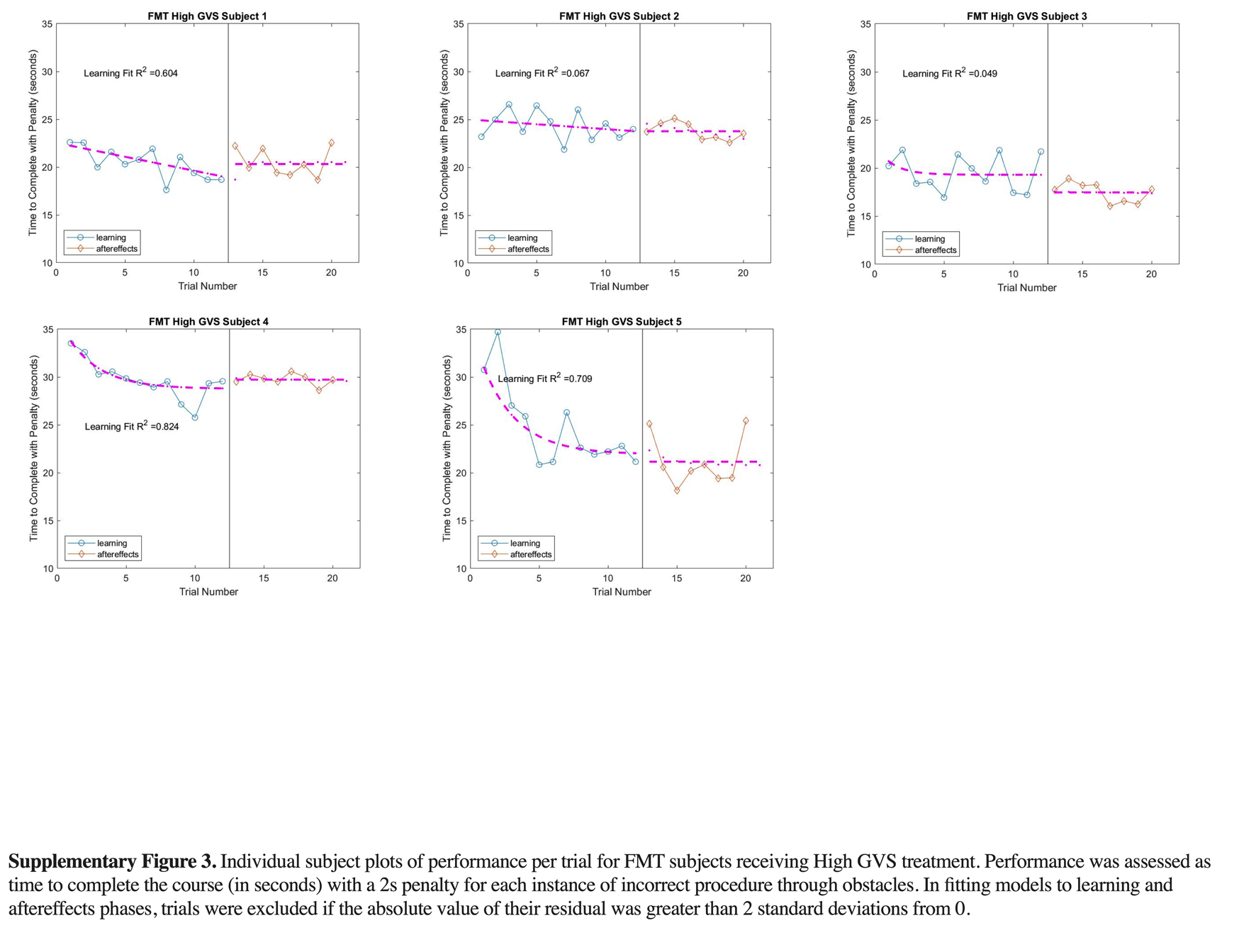

Supplement: Supplementary file 3 [file Image_3.TIFF]

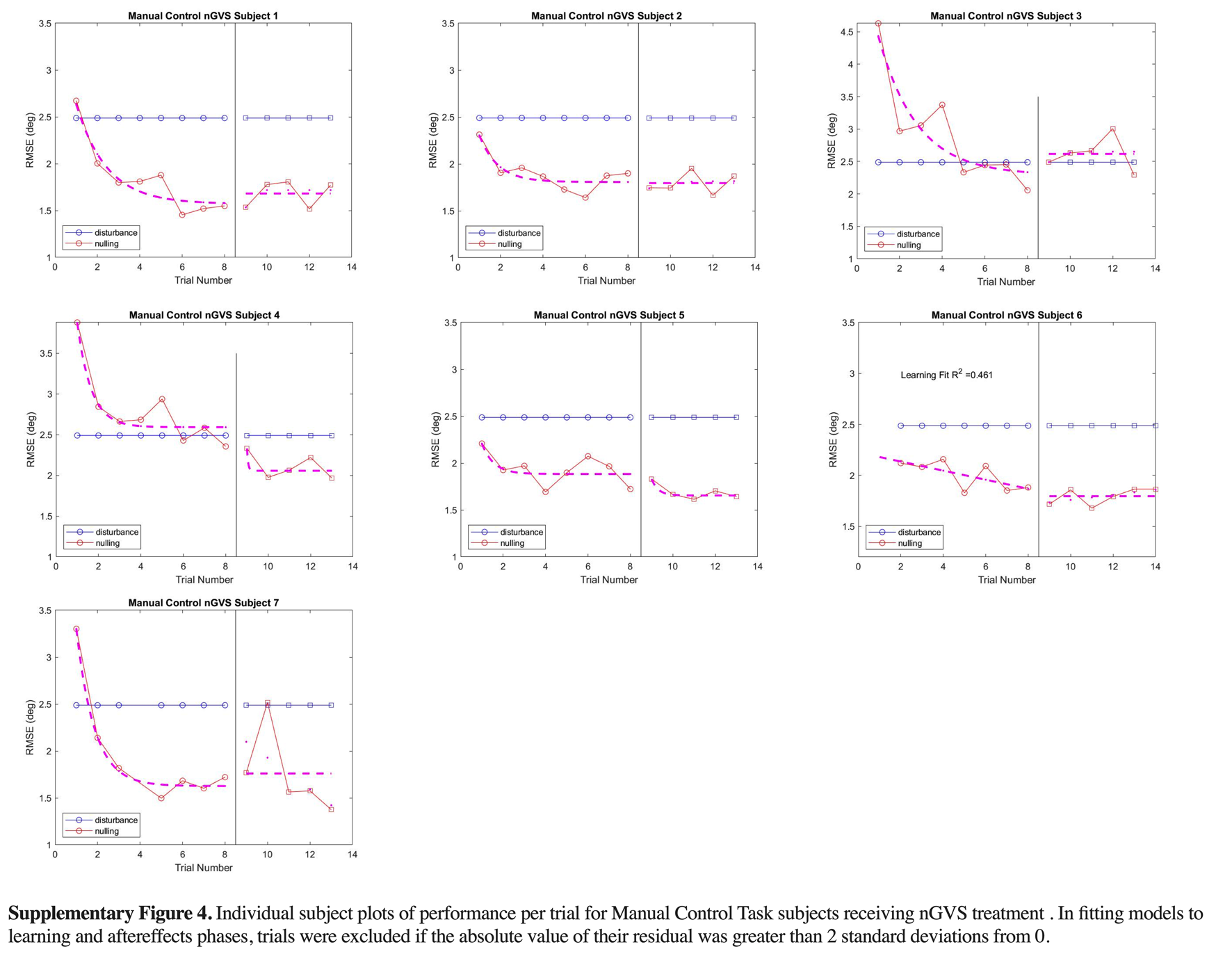

Supplement: Supplementary file 4 [file Image_4.TIFF]

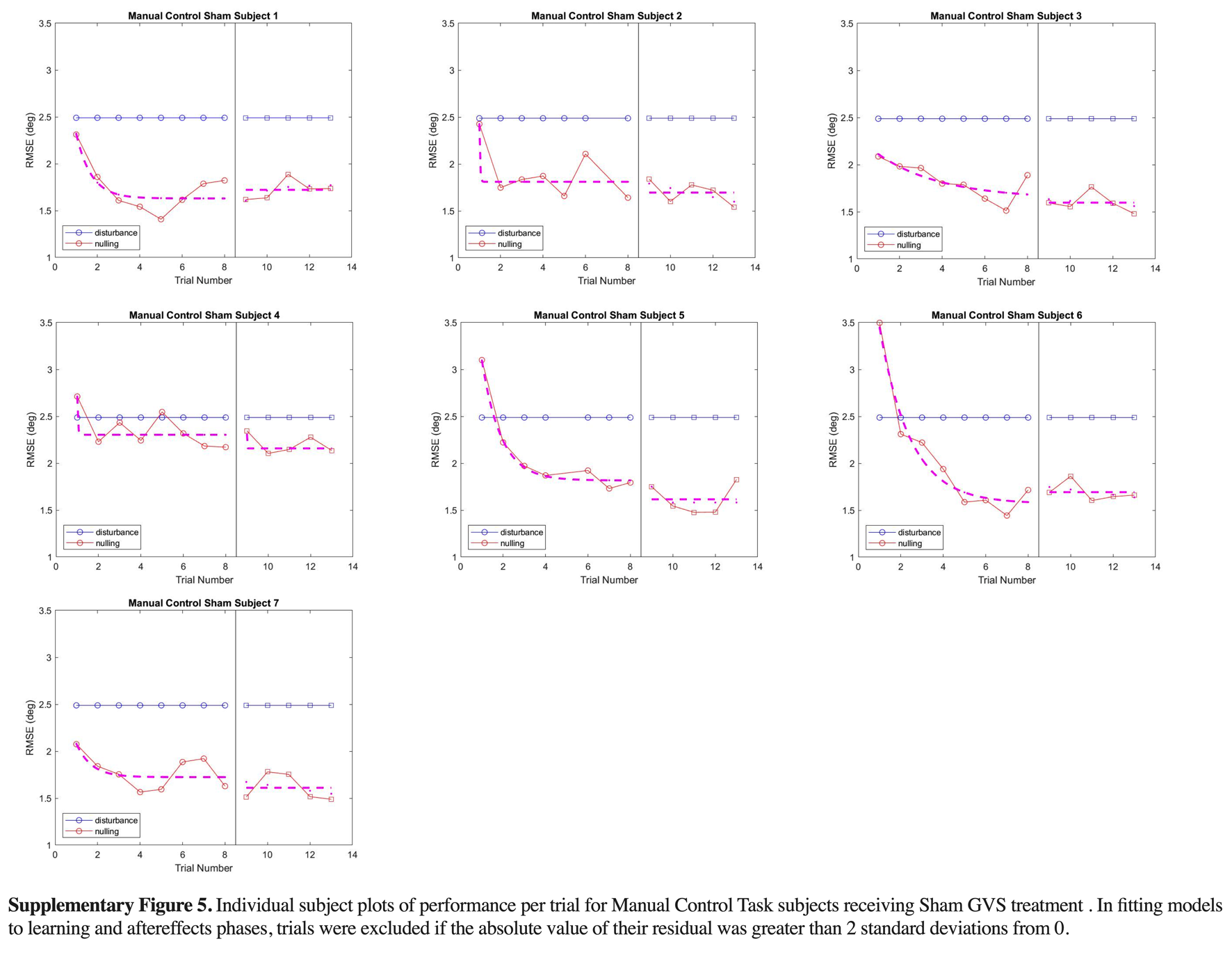

Supplement: Supplementary file 5 [file Image_5.TIFF]

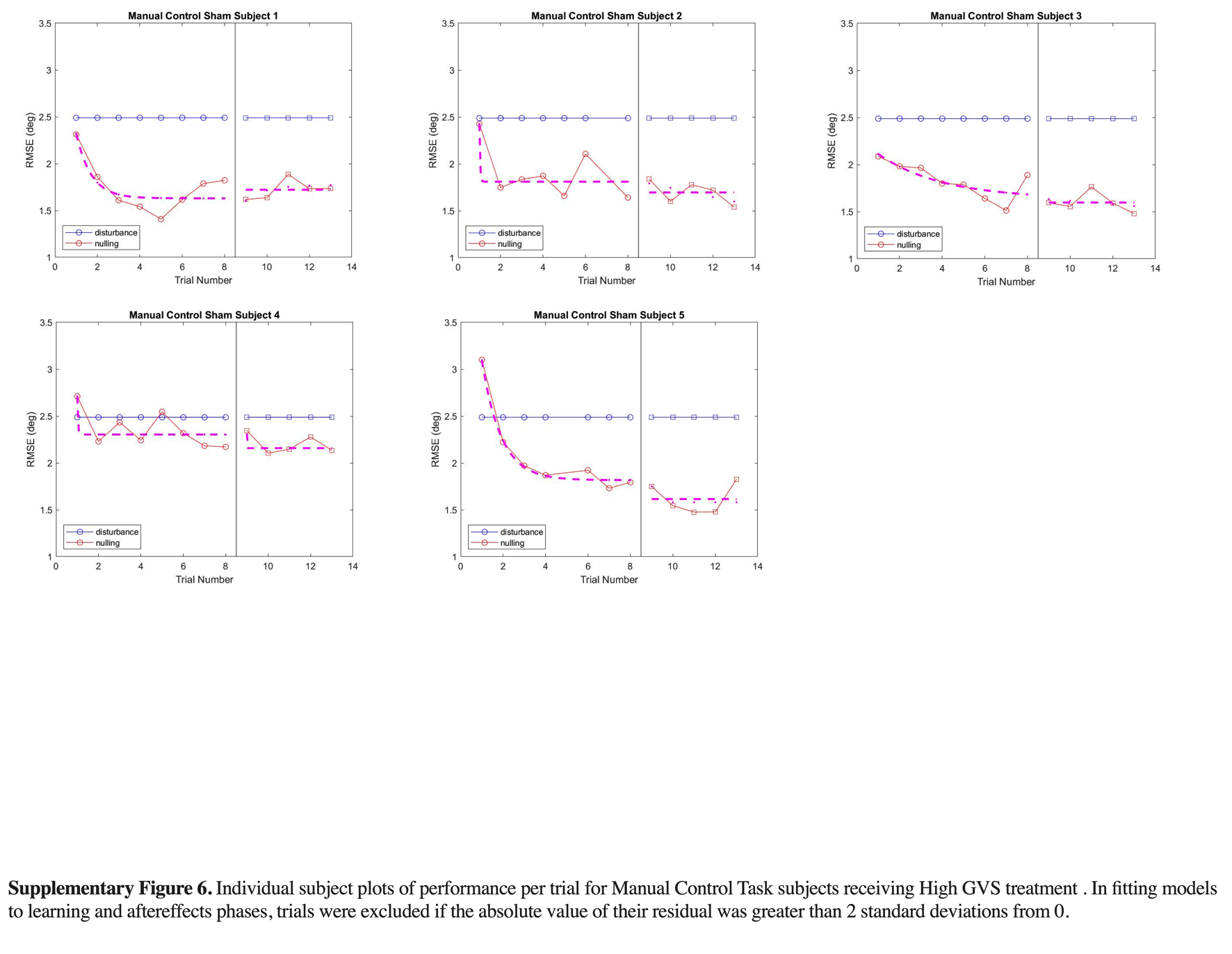

Supplement: Supplementary file 6 [file Image_6.TIFF]
